# Antibacterial Activity and Cytotoxicity of Gold (I) and (III) Ions and Gold Nanoparticles

**DOI:** 10.4172/2167-0501.1000199

**Published:** 2015-12-20

**Authors:** TP Shareena Dasari, Y Zhang, H Yu

**Keywords:** Gold nanoparticle, Gold (I) and (III) ions, Antibacterial effect, Cytotoxicity

## Abstract

Gold nanoparticles (AuNPs) and gold ion complexes have been investigated for their antibacterial activities. However, the majority of the reports failed to disclose the concentration of free Au(I) or Au(III) present in solutions of AuNPs or gold ion complexes. The inconsistency of antibacterial activity of AuNPs may be due to the effect of the presence of Au(III). Here we report the antibacterial activity of Au(I) and Au(III) to four different bacteria: one nonpathogenic bacterium: *E. coli* and three multidrug-resistant bacteria: *E. coli, S. typhimurium DT104*, and *S. aureus*. Au(I) and Au(III) as chloride are highly toxic to all the four bacteria, with IC_50_ of 0.35 – 0.49 µM for Au(III) and 0.27–0.52 µM for Au(I).The bacterial growth inhibition by both Au(I) and Au(III) increases with exposure time and is strongly affected by the use of buffers. The IC_50_ values for Au(I) and Au(III) in different buffers are HEPES (0.48 and 1.55 µM) > Trizma (0.41 and 0.57 µM) > PBS (0.14 and 0.06 µM). Bacterial growth inhibition by AuNPs is gradually reduced by centrifugation-resuspension to remove residual Au(III) ion present in the crude synthetic AuNPs. After 4 centrifugations-resuspensions, AuNPs become non-toxic. In addition, both Au(I) and Au(III) are cytotoxic to skin keratinocyte and blood lymphocyte cells. These results suggest that Au(I) and Au(III) in pure or complex forms may be explored as a method to treat drug-resistant bacteria, and the test of AuNPs toxicity must consider residual Au(III), exposure time, and the use of buffers.

## Introduction

Gold nanoparticles (AuNPs) have attracted considerable interests for fundamental and applied research. As AuNP applications continue to increase, growing human safety concerns are gaining attention [1–9]. It was pointed out that the toxic effects of AuNPs are complex due to co-existing chemicals, such as the presence of citrate and Au(III) ions during the photomutagenecity test of AuNPs [10]. Gold nanorods are toxic to human skin cells due to the surface coating chemical CTAB (cetyltrimethylammonium bromide), but not the gold nanorods [11– 13]. CTAB alone is toxic to cells at sub-micromolar concentrations. The free CTAB molecules in gold nanorod solutions may be a result of inadequate purification and desorption of surface CTAB from the gold nanorods. Thus, it was suggested that proper control experiments must be carried out when studying the toxicity of AuNPs.

Studies on the antimicrobial effects of AuNPs are summarized in a recent review article [14]. Among the 70 plus reports on the antimicrobial effect of AuNPs, at least eight of them suggested that AuNPs are either not or very weakly antibacterial, while the others reported various degrees of antibacterial activity. These discrepancies in the antibacterial activities of AuNPs may be due to several factors: 1) the use of surface coating agents on AuNPs including antibiotics, 2) the test methods, and 3) residual Au(III). Since most AuNPs preparation methods involve chemical reduction of Au(III) salts in aqueous, organic, or mixed phases in the presence of reductants and surface-coating agents such as antibacterial compounds, the AuNPs solution is a mixture since some of them are used without proper purification.

Residual Au(III) ions could cause false antibacterial test results [10,14,15]. In fact, gold ions as organic complexes have been of interest as antimicrobial agents [14,16–18]. Many reported that Au(I) and Au(III) complexes with organic ligands are effective against a wide variety of microorganisms [16–23] including the review article by Gilisci and Djuran that summarized the antibacterial activity of Au(I) and Au(III) complexes [17]. Au(I) and Au(III) complexes are soluble in organic solvents, but their lack of aqueous solubility limits the potential use as antibacterial or therapeutic agents.

We hypothesize that 1) Au(I) or Au(III) is antibacterial; 2) synthetic AuNPs, if followed with proper removal of residual Au(III) ions, are weakly or not toxic to bacteria; 3) the antibacterial activity of Au(III) and Au(I) is dependent on the exposure time and the use of different buffers. Thus, we evaluated the biological activity of Au(I) and Au(III) on four bacteria: one nonpathogenic bacterium *E. coli* and three multidrug-resistant bacteria *E. coli, S. typhimurium* DT-104, and *S. aureus*; and two human cell lines: a skin keratinocyte and a blood lymphocyte cell line. The effect on the antibacterial activity by treatment time and the use of buffers (PBS, Trizma, and HEPES) were evaluated. The antibacterial effect of AuNPs was studied upon centrifugations to remove residual Au(III) from the synthesis.

## Materials and Methods

### Materials

Chloroauric acid (HAuCl_4_, 99%, a form of Au(III) in solution) was purchased from Sigma-Aldrich and used without further purification. Gold (I) chloride (AuCl) was purchased from Strem Chemicals (Newburyport, MA). AuCl, due to its limited water solubility, was first suspended in nanopure water through sonication, and then the undissolved AuCl was filtered through 0.2 µm filter (Corning Incorporated, NY). The filtered solution had a final Au(I) concentration of 2.87 mM determined by ICP-MS (Varian Model No. 820-MS). Bacteria and cell lines used in this study include non-pathogenic *E. coli* (BAA-1431), multidrug-resistant *E. coli* (BAA-1161), multidrug-resistant *S. typhimurium* DT-104 (ATCC 700408), multidrug-resistant *S. aureus* (MRSA, BAA-44), and the blood lymphocyte cell line, TIB-152, were purchased from American Type Culture Collection (ATCC) (Manassas, VA). The HaCaT keratinocyte, a transformed human skin cell line, was obtained from Dr. Norbert Fusenig of the Germany Cancer Research Center (Heidelberg, Germany). RPMI-1640 medium was purchased from ATCC (Manassas, VA). Trypsin EDTA solutions were purchased from Cambrex Bio Science (Walkersville, MD). Tryptic Soy Broth (TSB) and Tryptic Soy Agar (TSA) used to grow the bacteria, and Trizma base (tris-(hydroxymethyl)aminomethane) and HEPES (4-(2-hydroxyethyl)-1-piperazineethanesulfonic acid) salts were purchased from Sigma-Aldrich (St. Louis, MO). Fetal bovine serum (FBS), Dulbecco’s Minimum Essential Medium (DMEM), penicillin/streptomycin, dimethyl sulfoxide (DMSO), phosphate buffered saline (PBS), and CellTiter 96^®^ AQ_ueous_ One Solution Cell Proliferation Assay (MTS) were purchased from Fisher Scientific (Houston, TX).

### Gold nanoparticle synthesis, purification, and characterization

**Figure 1:**
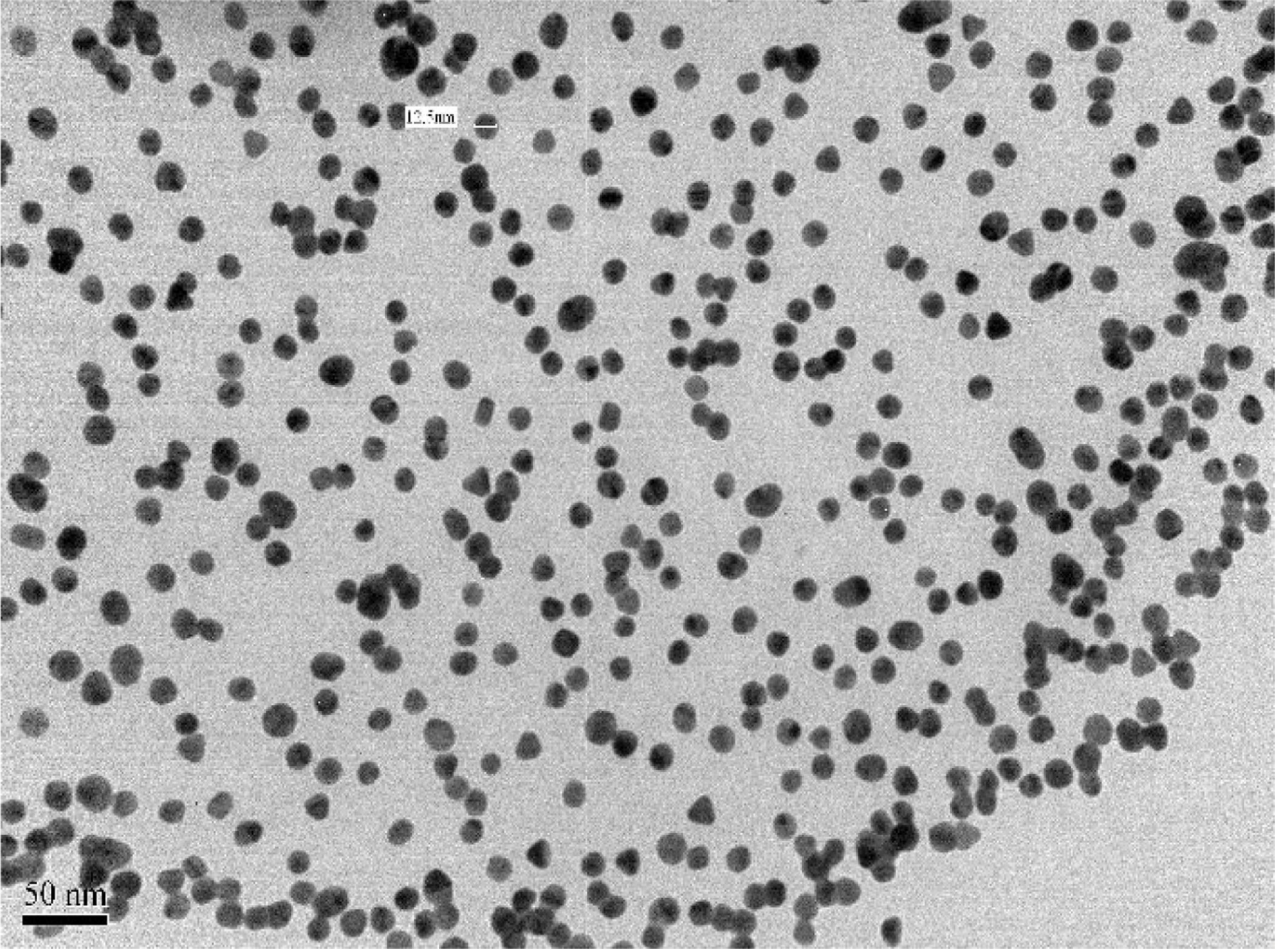
TEM image of the synthesized AuNPs with size range of 15–25 nm.

AuNPs were prepared by adding 5 mL of HAuCl_4_ (10 mM) solution and 5 mL of 38.8 mM sodium citrate (Na_3_C_6_H_5_O_7_) to 45 mL of boiling H_2_O. Further heating (100°C) up to 20 min caused the solution to turn from yellow to wine red [24]. The solution was centrifuged at 5000 rpm for 2 h at 20°C and the resulting AuNPs pellet was washed with 5 mL of 0.1 mM sodium citrate buffer to remove residual Au(III) ions and citrate ions. This procedure was repeated 1–4 times to eliminate residual Au(III) ions. All experiments were carried out with the AuNPs ranging 15–25 nm (Figure 1). The characterization of AuNPs was carried out by UV-Vis and TEM (JEM 1011, Joel Inc.) and AuNP concentration (0.97 mM) was determined using UV-Vis as reported previously [10,13].

### Bacterial growth inhibition assay with Au(I), Au(III), and synthesized AuNPs

Bacterial growth inhibition was carried out using the spread plate counting method as reported before [25]. *E. coli, Salmonella* DT-104, and *S. aureus* were cultured in TSB growth medium at 37°C, 200 rpm for 10–12 h in a shaker incubator. The bacterial cultures were centrifuged at 3000 rpm for 20 min, and residual bacteria were resuspended in sterilized physiological saline (0.85% NaCl). Bacterial density was adjusted to 3 × 10^8^ cells/mL in PBS. The final exposure concentrations for Au(III) were 0, 0.01, 0.03, 0.1, and 0.3 µM, and for Au(I) were 0, 0.1, 0.3, 1, and 3 µM. The final concentrations of Au(III) and Au(I) for time dependent experiments were 0.1 and 1 µM, respectively. Au(III) and Au(I) solution at different concentrations were combined with the cultured bacteria and placed in a shaker incubator with continuous agitation at 200 rpm for 2 h or a designate time at 25°C. The time dependent samples (100 µL) were transferred onto TSA plates after 30, 60, 90, 120, 150 and 180 min of shaking. The transferred samples were evenly spread onto the pre-prepared agar plates, and all plates were inverted and incubated at 37°C for 24 h. For the test with different buffers, PBS (pH 7.4), Trizma (pH 7–9) and HEPES (pH 7.4) buffers were used at the final concentration of 1 mM. In addition, we tested the inhibition of bacterial growth by AuNPs with 0–4 centrifugations at 2 h exposure.

### Cytotoxicity assay

HaCaT cells were grown in complete medium (DMEM, 10% FBS, and 1% Fungizone, penicillin/streptomycin) in 25 cm^2^ cell culture flasks. Cells were cultured in a humidified incubator with 5% CO_2_ at 37°C. After the cells grew to confluence, they were detached by 25% trypsin/DTA and diluted to 3×10^5^ cells/mL by their respective complete media as reported before [26]. A 200 µL cell suspension in complete medium was added to each well of a 96-well plate and incubated under 5% CO_2_ at 37°C for 24 h for cell adhesion. TIB-152 cells were grown in complete medium (RPMI-1640, 10% FBS) in 75 cm^2^ culture flasks in the incubator until 1×10^6^ cells/mL was achieved, and they were then centrifuged and resuspended in RPMI-1640 medium. After incubation, the supernatant was pipetted, and the adherent cells were washed with 1× PBS before exposed to Au(III). Then a total of 90 µL of DMEM (for HaCaT) or EMEM (for TIB-152) and 10 µL of Au(III) at desired concentrations were added to each well. A total of 3 wells were used for the test at each concentration. After 2 h or 24 h of treatment, cell viability was determined by the MTS assay: 20 µL MTS solution was added directly to each well after treatment and the absorbance was read at 490 nm using a 96-well Multiskan Ascent Plate Reader with Ascent software. The control test was performed with PBS buffer.

### Statistical analysis

At least three independent experiments were carried out for each point. All statistical analyses were performed using the SAS 9.3 software. Significance was determined with Generalized Linear Model and Tukey’s test to distinguish the differences between the variables for bacterial growth inhibition assays. Significance was determined with Generalized Linear Model (Duncan) for cytotoxicity assays. The significance level was defined as p < 0.05. IC_50_ values were determined using SPSS software (IBM^®^ SPSS^®^ Statistics, Version 22).

## Results and Discussion

### Concentration and time-dependent inhibition of *Salmonella* DT-104, *E. coli* and MRSA growth by Au(I) and Au(III)

**Figure 2:**
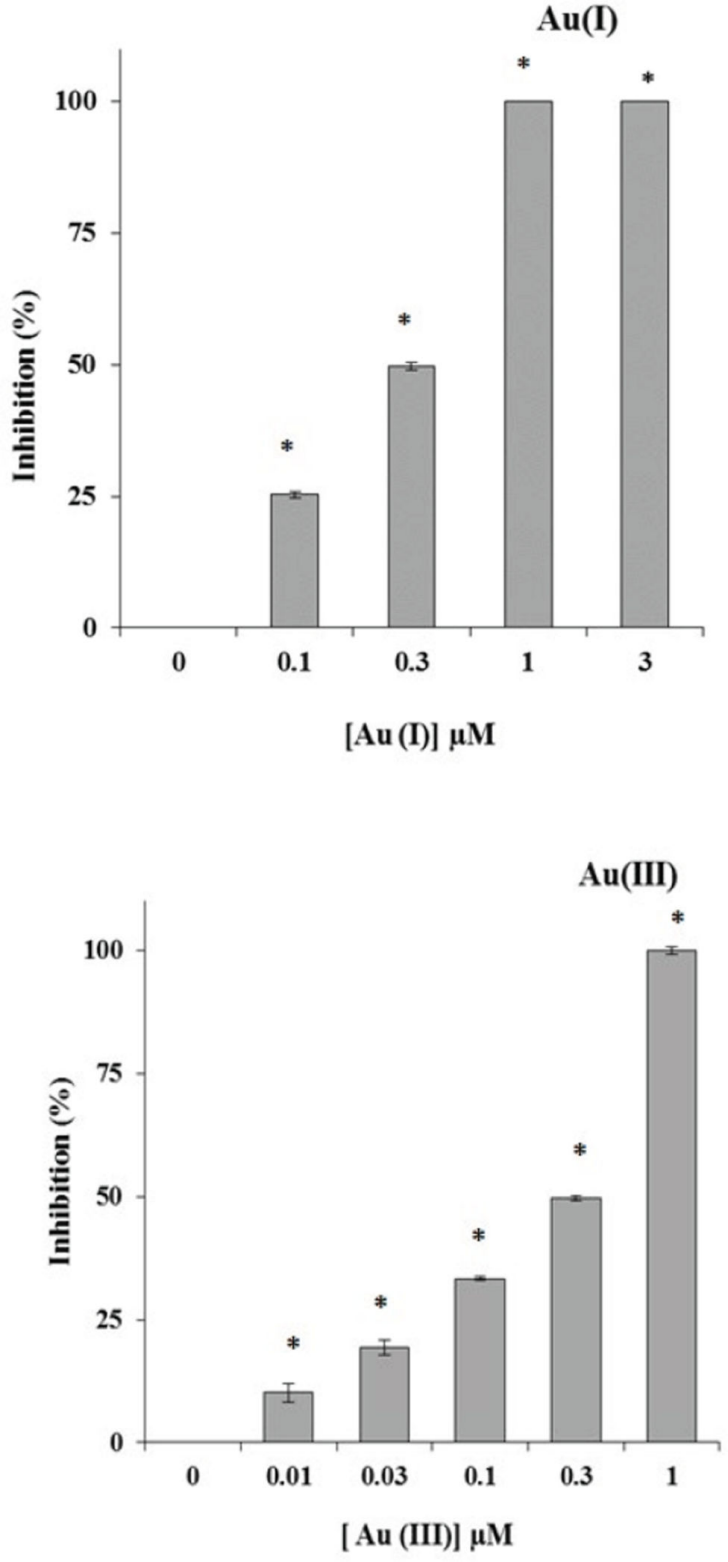
Concentration dependent growth inhibition by Au(I) and Au(III) ions on multi-drug resistant *E. coli* (BAA-1161). Error bars are standard deviations (n=3). * Denotes significant with p < 0.05.

We tested bacterial growth inhibition of the four selected bacteria after exposure to Au(I) or Au(III) for 2 h. Figure 2 shows the concentration dependent inhibition of the multi-drug resistant *E. coli* (BAA-1161) growth after exposure to 0.1, 0.3, 1, and 3 µM of Au(I) or 0.01, 0.03, 0.1, 0.3, and 1 µM of Au(III). The other three bacteria show the same pattern of inhibition (data not shown). These results confirm that Au(III) and Au(I) are toxic to all the four bacteria even at very low concentrations.

From the concentration-dependent inhibition data, IC_50_ values were determined by SPSS and are listed in table 1. Among the four bacteria, the Gram positive MRSA has the highest IC_50_ values (0.49 µM for Au(I) and 0.52 µM for Au(III)), while the three Gram negative bacteria have similar IC_50_ values 0.35–0.39 µM for Au(I) and 0.27–0.36 µM for Au(III). The difference in toxicity for the Gram positive and the Gram negative bacteria could be due to differences in cell wall structure. The bacterial cell wall plays a vital role in resistance or susceptibility [27]. There is very little difference in IC_50_ values between Au(I) and Au(III) for *E. coli:* 0.38 µM versus 0.34 µM for BAA-1431 and 0.35 versus 0.36 for BAA-1161, and MRSA, 0.49 µM versus 0.52 µM, respectively. However, Au(III) is more toxic to the *Salmonella* DT104 than Au(I) (IC_50_ of 0.27 µM versus 0.39 µM). The reasons for these differences in toxicity need further investigation.

Wang et al first reported the photomutagenicity of Au(III) on *S. typhimurium* TA102 and pointed out that Au(III) is also toxic to the bacterium at a concentration at 1 µM [10]. Nam et al very recently reported an extensive study of Au(III) toxicity to a variety of microorganisms including bacteria in 2014 [15]. Due to the toxicity of Au(I) and Au(III), the tests of antibacterial activity of AuNPs as well as Au(I) and Au(III) complexes have to consider the possible presence of residual Au(I) and Au(III). Without proper purification to remove the residual Au(I) and Au(III), it may lead to erroneous results.

**Figure 3:**
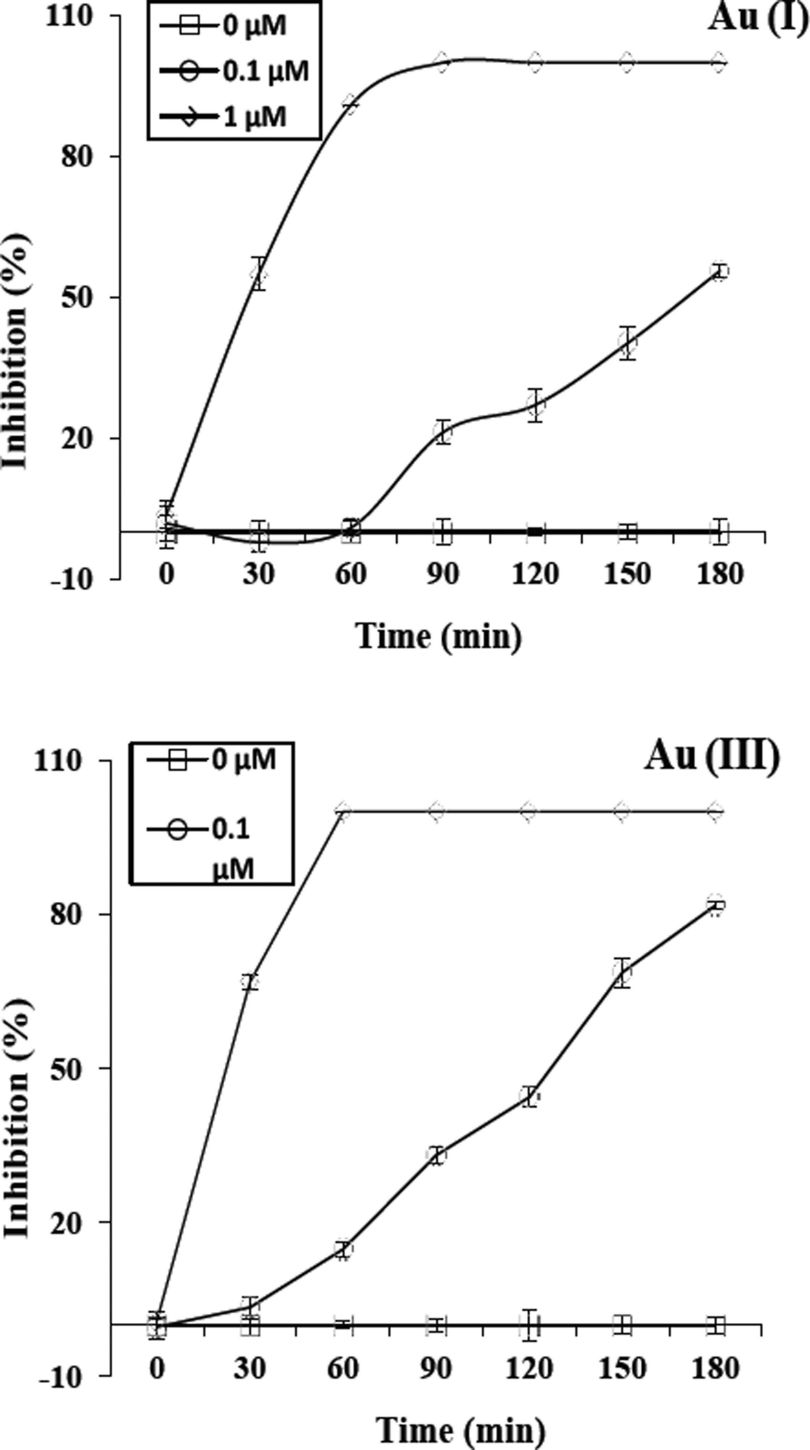
Time-dependent growth inhibition by Au(I) and Au(III) against nonpathogenic *E. coli* at concentrations 0, 0.1 and 1µM.

Time-dependent inhibition experiments were carried out to see how exposure time affects the toxicity of Au(I) and Au(III). Figure 3 shows the inhibitory effects on *E. coli* after exposure to Au(I) and Au(III) up to 3 h at two different concentrations (0.1 and 1 µM). At 1 µM, the percent of bacterial growth inhibition increases from exposure time of 30 min to 60 min, and it reaches near 100% inhibition at longer than 60 min. At 0.1 µM, the inhibition is not detectable at 30 and 60 min exposure, but continues to increase from 90 to 180 min (3 h). It seems not reaching maximum inhibition even at 3 h. Nam et al examined much longer exposure time of 72 h [15]. They found that Au(III) continues to be toxic for some bacteria at longer exposure times. This clearly shows that the toxicity of Au(I) and Au(III) to these bacteria is exposure time dependent and suggests that exposure time must be factored in when conducting similar tests.

### Effect of buffers on the growth inhibition of Au(I) and Au(III) to nonpathogenic *E. coli*

**Figure 4:**
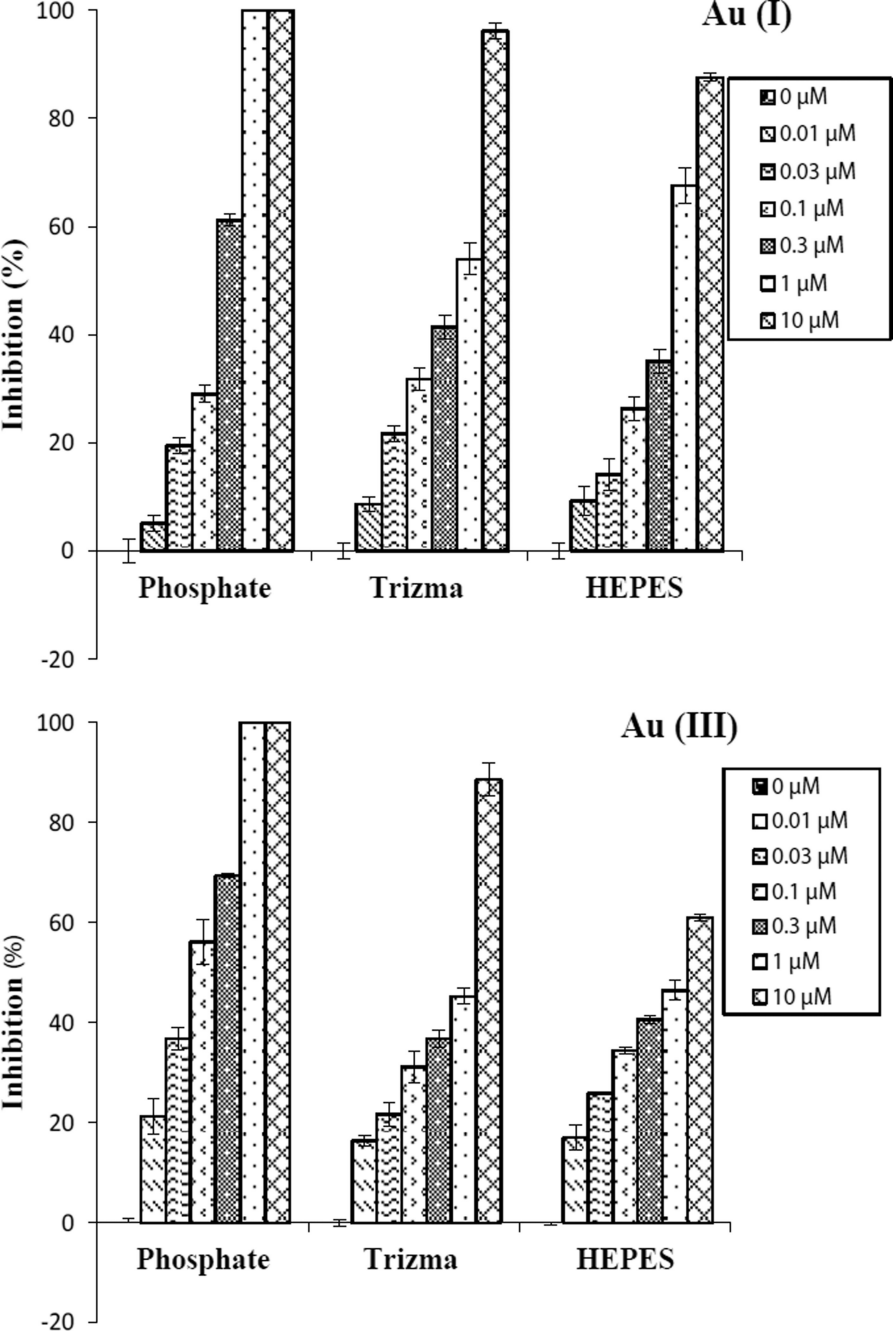
Growth inhibition of nonpathogenic *E. coli* by exposure to Au(I) and Au(III) at 0, 0.01, 0.03, 0.1, 0.3, 1 and 10 µM for 2 h in PBS, Trizma and HEPES.

PBS, Trizma, and HEPES are three commonly used buffers. The effect of using these buffers (0.01, 0.03, 0.1, 0.3, 1 and 10 µM) on the toxicity of Au(I) and Au(III) to the nonpathogenic *E. coli* was tested with 2 h exposure time (Figure 4). The IC_50_ values were determined using SPSS, and they are listed in table 2: 0.14, 0.41, and 0.48 µM for Au(I) and 0.06, 0.57 and 1.55 µM for Au(III), in PBS, Trizma, and HEPES buffers, respectively. Complete (100%) inhibition was observed for bacteria exposed to Au(III) and Au(I) at 1 µM in PBS buffer, whereas in Trizma and HEPES buffers at 10 µM of Au(III) and Au(I), some bacteria are still viable. Therefore, the buffers used for this type of test have a profound effect on their toxicity. It is more profound for Au(III), where the IC_50_ in HEPES is 24 times of that in PBS. We believe that the use of buffers has an effect on how Au(I) and Au(III) behave in solution and how they interact with the bacterial cells. More advanced studies are needed to understand the effect of these buffers on the antibacterial effect of gold ions.

### Effect of centrifugation-resuspension on the growth inhibition of AuNPs to nonpathogenic *E. coli*

**Figure 5:**
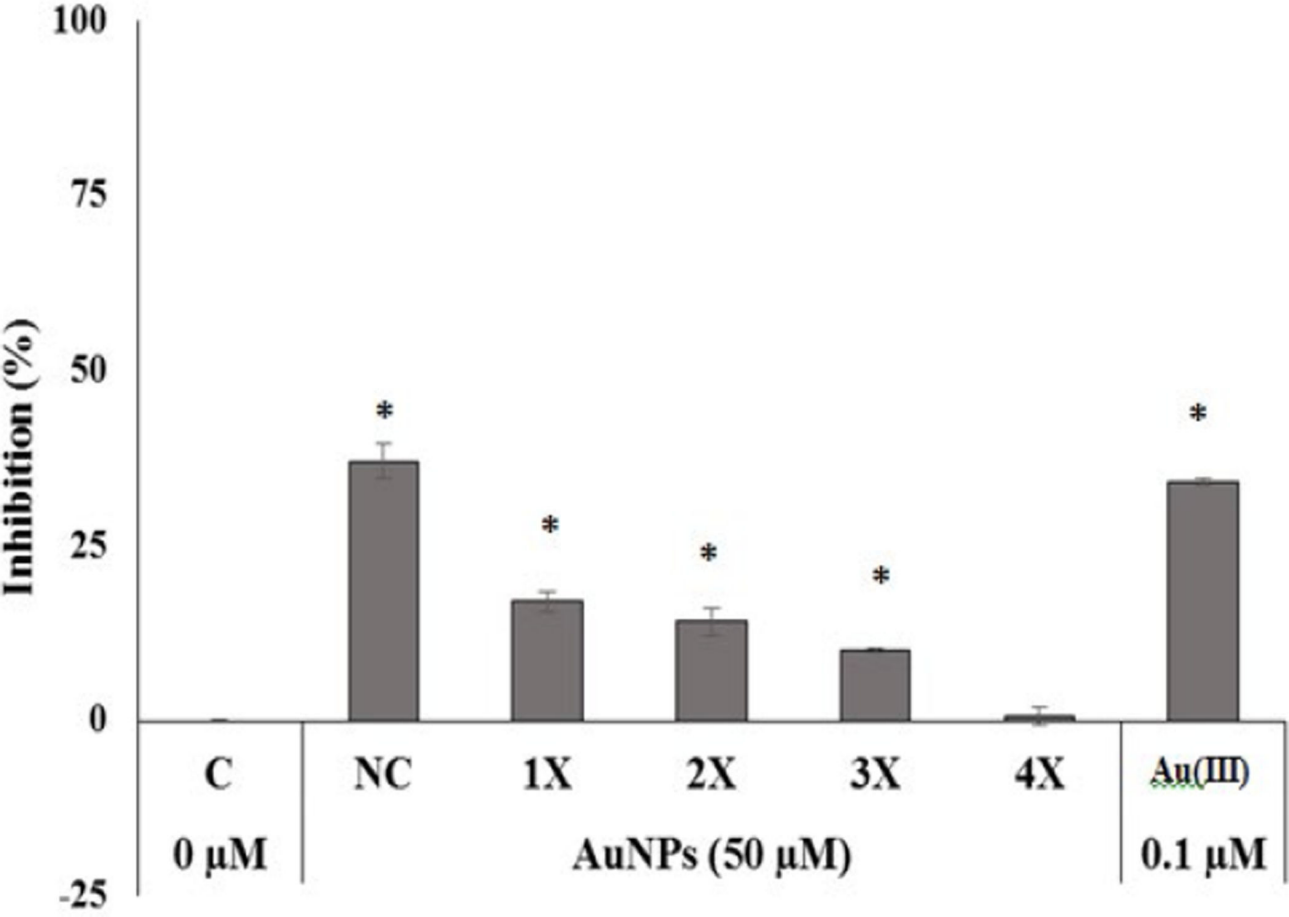
Effect of centrifugations of AuNPs on growth inhibition of non-pathogenic *E. coli* by AuNPs. Control (C), Non-centrifuged (NC), 1× (Centrifuge 1), 2× (Centrifuge 2), 3× (Centrifuge 3) and 4× (Centrifuge 4). * Denotes significant with p < 0.05.

*E. coli* growth inhibition by 50 µM of AuNPs (non-centrifuged) was compared with centrifuged (1–4 centrifugations-resuspensions) at 5000 rpm for 2 h each at 20°C. There is a 37% inhibition with non-centrifuged AuNPs, but the bacterial growth inhibition decreases to 17, 14, 10, and 1%, respectively, upon 1–4 centrifugations (Figure 5). This demonstrates that AuNPs, once the Au(III) is completely removed, do not inhibit the growth of *E. coli*. Based on figure 2 and the calculated IC_50_ values of 0.34 µM for Au(III), the percent of inhibitions of 37, 17, 14, 10 and 1% correspond to Au(III) concentrations of 0.11, 0.05, 0.04, 0.02 and 0.001 µM. This demonstrates that residual Au(III) present in AuNPs was removed through 3–4 centrifugations. Without purifications, false toxicity result could occur. We tried to obtain the accurate Au(III) concentrations in the supernatant after centrifugations, but the use of an ICP-MS, which determines total gold concentration, did not yield accurate Au(III) concentration due to the small amount of AuNPs retained in the supernatant.

### Cytotoxicity of Au(III) to skin (HaCaT) and blood (TIB-152) cells

**Figure 6:**
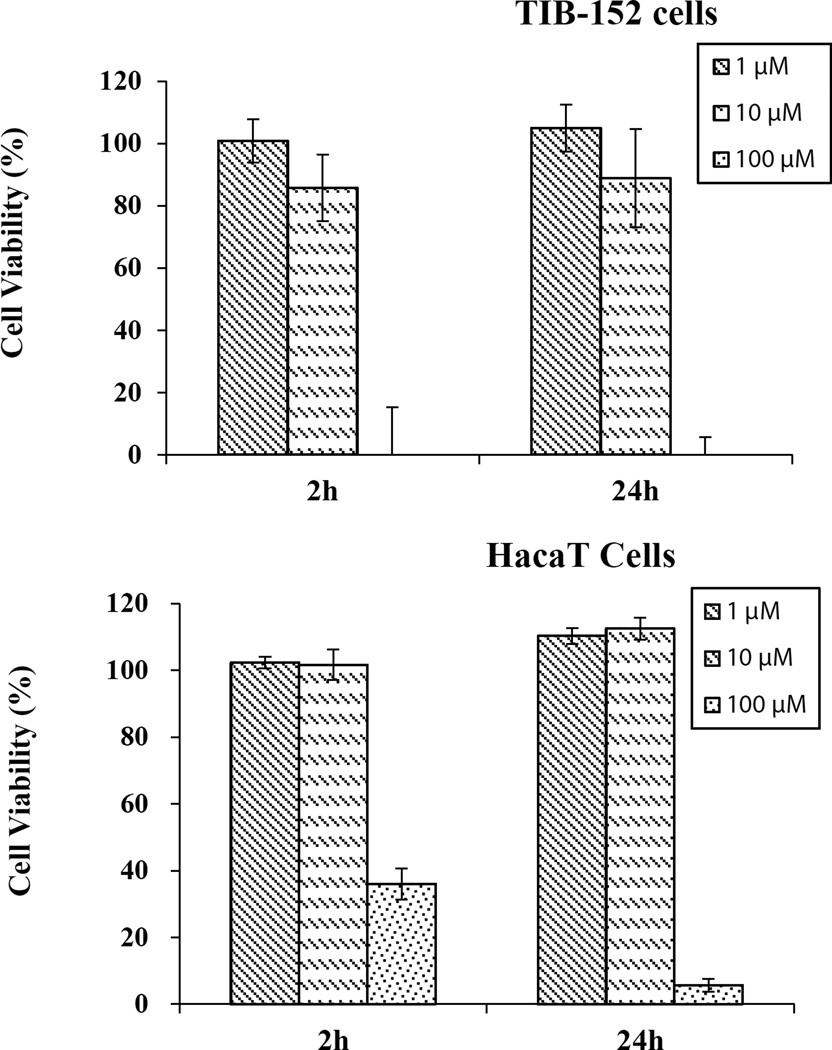
Percent cell viability of HaCaT and TIB-152 cells after 2 h and 24 h treatment with Au(III).

Since Au(III) is antibacterial, we want to know if it is also cytotoxic to human cells: skin keratinocyte (HaCaT) and blood lymphocyte (TIB-152) cells. The reason these two cell lines were chosen was because they are the likely targets during skin exposure and blood transport of environmental toxins. The cells (HaCaT and TIB152) were exposed to Au(III) at 1, 10 and 100 µM for 2 h and 24 h. The cell viability was determined by MTS assay (Figure 6). Au(III) is not toxic at 1 and 10 µM at both exposure times, but it is toxic at 100 µM for both cell lines with cell viabilities of 36% and 8% against HaCaT cells at 2 h and 24 h exposure times, respectively. Whereas 100 % inhibition was observed at 24 h exposure time against TIB-152 cells at 100 µM Au(III) (Figure 6).

## Concluding Remarks

This work clearly demonstrates that both Au(I) and Au(III) ions are strongly antibacterial against all four tested bacteria: one nonpathogenic *E. coli* and three multidrug resistant bacteria: *E. coli, S. typhimurium DT104*, and *S. aureus (MRSA)*. Nonlinear dose-dependent growth inhibition is observed and the antibacterial effect of Au(I) and Au(III) ions vary slightly with the type of bacteria. The length of treatment has a significant effect on the antibacterial effect of Au(III) and Au(I). For sub-IC_50_ concentrations, both Au(I) and (III) ions continue to inhibit the bacterial growth even after 3 h. The use of buffer plays a significant role in altering the antibacterial activity of both Au(III) and Au(I). The antibacterial activity is strongest in PBS, followed by Trizma and HEPES. Centrifugation of AuNPs 1–4 times to remove residual Au(III) ions reduces the antibacterial effect of “AuNPs”, suggesting that AuNPs alone do not inhibit bacteria growth for the four bacteria tested. Au(III) ions are also toxic to HaCaT and TIB152 cells at high concentrations (100 µM). The antibacterial effects of Au(III) and Au(I) may be further explored for medical purposes, especially against those three multi-drug resistant bacteria.

On the other hand, test of biological effects of nanoparticles, due to the fact that nanoparticles in solution are often a chemical mixture [26], careful controls and purifications must be carried out. Our results demonstrate that the antibacterial effect of AuNPs is strongly dependent on the number of centrifugations to remove excess Au(III), the use of buffers, the exposure time, the type of bacteria and test method. This might explain the discrepancies in the literature concerning the antibacterial effect of AuNPs since there was no mentioning about purification of the AuNPs in most of the reports. For some of the antibacterial results of the gold (I) and (III) organic complexes, one may need to investigate whether free gold ions played a role [14].

## Figures and Tables

**Table 1:** IC_50_ for bacterial growth inhibition by Au(I) and Au(III). Only the IC_50_ against MRSA is significantly different against the other three bacteria (p < 0.05).

	Au(I) (µM)	Au(III) (µM)
*E. coli* (BAA-1431)	0.38	0.34
*E. coli* (BAA-1161)	0.35	0.36
*Salmonella* DT-104 (ATCC 700408)	0.39	0.27
MRSA (BAA-44)	0.49 (*)	0.52 (*)

**Table 2:** IC_50_ values for Au(I) and Au(III) against nonpathogenic *E. coli* in PBS, Trizma and HEPES buffers. Only the values in PBS are significantly different (p < 0.05)

	Au(I) (µM)	Au(III) (µM)
PBS	0.14 (*)	0.06 (*)
Trizma	0.41	0.57
HEPES	0.48	1.55
